# Psychometric validation of the EORTC QLQ-HCC18 in patients with previously treated unresectable hepatocellular carcinoma

**DOI:** 10.1007/s11136-021-02992-1

**Published:** 2021-09-13

**Authors:** Daniel Serrano, Lauren Podger, Gisoo Barnes, James Song, Boxiong Tang

**Affiliations:** 1 Pharmerit (An Open Health Company), 4350 East-West Highway, Suite 1100, Bethesda, MD 20814 USA; 2 BeiGene, Ltd, San Mateo, CA USA

**Keywords:** Hepatocellular carcinoma, Patient-reported outcomes, Health-related quality of life, Psychometric analysis, Classical test theory, Responsiveness, Meaningful change, EORTC QLQ-HCC18

## Abstract

To demonstrate the measurement properties of the European Organization for Research and Treatment of Cancer Quality of Life Questionnaire Hepatocellular Carcinoma 18-question module (EORTC QLQ-HCC18) within a previously treated, unresectable hepatocellular carcinoma (HCC) clinical trial population that was distinct from the published QLQ-HCC18 validation population. Analyses were conducted using data from BGB-A317-208, an open label, international, clinical trial assessing efficacy and safety of the monoclonal antibody tislelizumab in adult HCC patients. The EORTC Quality of Life Questionnaire Core 30 (EORTC QLQ-C30) and QLQ-HCC18 instruments were assessed at baseline and weeks 3 and 9 follow-up visits. Per US Food and Drug Administration guidance, psychometric validation of the QLQ-HCC18 included reliability (internal consistency and test–retest), construct validity (convergent and discriminant validity and known-groups validity), ability to detect change, and meaningful within-patient change (MWPC). Known-groups validity and MWPC analyses were also stratified on several pre-defined subgroups. A total of 248 patients were included. Only the QLQ-HCC18 fatigue, nutrition, and index domains demonstrated acceptable internal consistency; acceptable test–retest reliability was found for fatigue, body image, nutrition, pain, sexual interest, and index domains. The QLQ-HCC18 fatigue domain achieved the pre-specified criterion defining acceptable convergent and discriminant validity for 13 of 16 correlations, whereas the index domain achieved the pre-specified criterion for 14 of 16 correlations. Clear differentiation of the QLQ-HCC18 change scores between improvement and maintenance anchor groups were observed for body image, fatigue, pain, and index domains, whereas differentiation between deterioration and maintenance anchor groups were observed for fever and fatigue domains. MWPC point estimates defining improvement for the QLQ-HCC18 fatigue and index domains were −7.18 and −4.07, respectively; MWPC point estimates defining deterioration were 5.34 and 3.16, respectively. The EORTC QLQ-HCC18 fatigue and index domains consistently demonstrated robust psychometric properties, supporting the use of these domains as suitable patient-reported endpoints within a previously treated, unresectable HCC patient population.

## Introduction

Hepatocellular carcinoma (HCC) is a substantial global health challenge that accounts for 85% to 90% of all reported cases of liver cancer and is the fourth most common cause of cancer-related death [1]. In addition, between 80 and 90% of people worldwide with HCC have comorbid hepatitis B virus (HBV) and/or hepatitis C virus (HCV) infection [2, 3]. The majority of HCC cases (> 80%) occur in Eastern Asia and sub-Saharan Africa, with typical incidence rates of > 20 per 100,000 individuals: China alone accounts for approximately 50% of both new HCC cases and HCC-related deaths worldwide [4, 5]. Southern European countries, such as Spain, Italy, and Greece, have higher incidence rates (10 to 20 per 100,000 individuals) in comparison to Northern Europe and the Americas [4, 5].

Patients with unresectable HCC represent a population with great unmet medical need, having a 5-year overall survival (OS) rate of 18% [6]. These patients often report symptoms (e.g., muscle cramps, pain, fatigue, sleep dysfunction) severe enough to affect their health-related quality of life (HRQoL) [7]. Furthermore, these symptoms affecting HRQoL have been found to correlate with shorter OS [7–10]. As a result, there has been a shift toward increased recognition of the need to assess HRQoL alongside traditional clinical outcomes in HCC trials [11]. Several different questionnaires have been employed to measure HRQoL in studies of HCC [7]; however, only the European Organization for Research and Treatment of Cancer Quality of Life Questionnaire Hepatocellular Carcinoma 18-question module (EORTC QLQ-HCC18) was developed specifically to assess symptom burden and impact on HRQoL in people with HCC [12, 13].

As it stands, there are limited published data demonstrating the measurement properties of the QLQ-HCC18 within an unresectable HCC population, as well as within specific subpopulations including viral hepatitis comorbidities (comorbid HBV and HCV versus no comorbidity), line of therapy (second- versus third-line or greater), and geographic region (Asia versus Europe). Furthermore, existing validation evidence supporting the robust psychometric properties of the QLQ-HCC18 was obtained within HCC populations distinct from that of the BGB-A317-208 trial population. Compared with the BGB-A317-208 population, most patients had early-stage disease (i.e., Barcelona Clinic Liver Cancer [BCLC] A) and previously underwent surgical treatment, ablation, or embolization [12, 13]. Very few patients in previous validation studies received systemic therapy, but all patients in this trial had received previous systemic therapy. Given these differences in the context of use, the objective of the current project was to validate the QLQ-HCC18 within the BGB-A317-208 trial population. In addition to the context of use motivation, there are currently no published thresholds of meaningful within-patient change (MWPC) for the QLQ-HCC18 as recommended under US Food and Drug Administration (FDA) draft guidance 3 [14]. Thus, following FDA guidance [14, 15], analyses of the QLQ-HCC18 were conducted to evaluate measurement properties (reliability, construct validity, ability to detect change, and MWPC) within this patient population.

## Methods

This validation study was conducted using BGB-A317-208 trial data. BGB-A317-208 (NCT0341989) was an open label, multicenter, international, phase 2 clinical trial assessing the efficacy and safety of tislelizumab, an investigational humanized immunoglobulin IgG4 monoclonal antibody with high affinity and binding specificity for programmed cell death protein-1 (PD-1) [16, 17] in patients with unresectable HCC. Enrolled patients received tislelizumab (200 mg) intravenously every three weeks for a total of three or more 21-day treatment cycles, followed by long-term safety and survival assessments.

The protocol, any amendments, and informed consent form were reviewed and approved by the Independent Ethics Committees or Institutional Review Board in conformance with Good Clinical Practice and applicable regulatory requirements. This study was conducted in accordance with sponsor procedures, which comply with the principles of Good Clinical Practice, International Council for Harmonisation of Technical Requirements for Pharmaceuticals for Human Use Guidelines, the Declaration of Helsinki, and local regulatory requirements. The consent forms were signed and dated by the patient or the patient’s legally authorized representative before his or her participation in the study. A copy of each signed consent form was provided to the patient or the patient’s legally authorized representative and all signed and dated consent forms were retained in each patient’s study file or in the site file.

### Patients

Patients were male and female adults (≥ 18 years of age), enrolled from international study sites, with histologically confirmed HCC that was not amenable to a curative treatment approach and who had received ≥ 1 line of systematic therapy for unresectable HCC. In addition, patients were BCLC stage C or B not amenable to locoregional therapy or relapsed after locoregional therapy, and not amenable to a curative treatment approach. Patients also had a Child–Pugh A classification. All patients were required to have an Eastern Cooperative Oncology Group (ECOG) performance status score of ≤ 1 [18].

### Measures

HRQoL was assessed using three patient-reported outcome (PRO) instruments: the European Organization for Research and Treatment of Cancer Quality of Life Questionnaire Core 30 (EORTC QLQ-C30), the corresponding HCC-specific module (QLQ-HCC18), and the EQ-5D-5L. These PROs were collected at baseline and the first day of treatment cycle 2 (week 3), then every other treatment cycle up to cycle 12 (week 36). At each treatment cycle visit, the PRO administration occurred prior to any clinical activities or dosing. For purposes of this psychometric analysis, only QLQ-HCC18 and QLQ-C30 results are reported (the EQ-5D-5L was not employed in validation).

The EORTC QLQ-C30 [19] is a validated generic HRQoL instrument for cancer patients comprises a global health status (GHS)/QoL scale (two items); five functional scales: physical functioning (five items), role functioning (two items), emotional functioning (four items), cognitive functioning (two items), social functioning (two items); as well as three symptom scales, and several single items: fatigue (three items), nausea and vomiting (two items), pain (two items), and dyspnea, insomnia, appetite loss, constipation, diarrhea, and financial impact (one item each) [20]. The functional and symptom items are rated on a 4-point Likert scale (with 1 = ‘not at all’ to 4 = ‘very much’), while the GHS items are rated on a 7-point Likert scale (with 1 = ‘very poor’ to 7 = ‘excellent’). A high score on the GHS and functional scales indicates high HRQoL and a high level of functioning, whereas a high score on the symptom scales and items indicates a high level of symptom severity. The two individual GHS items were used as concurrent validators. The GHS scale of the QLQ-C30 was used as the PRO anchor variable in test–retest reliability, ability to detect change, and meaningful within-patient change analyses.

The EORTC QLQ-HCC18 [21] measures HCC-specific symptoms and HRQoL. The instrument is an 18-item scale, consisting of six symptom scales and two single items: fatigue (three items), body image (two items), jaundice (two items), nutrition (five items), pain (two items), fever (two items), sexual interest (one item), and abdominal swelling (one item). Scores are based on a 4-point Likert scale (with 1 = ‘not at all’ to 4 = ‘very much’); scaled scores for each domain ranged from 0–100 with a higher score indicating worse symptoms. In addition, an overall QLQ-HCC18 index score was defined to provide an overall characterization of all domains/items. The index score was calculated as the average of all non-missing QLQ-HCC18 scales [9]. Index scores ranged from 0–100, with a higher score indicating overall worse symptoms. Reporting of fatigue and index scores was prioritized in this validation exercise because these domains are important for the assessment of PRO-based clinical significance in the BGB-A317-208 trial. Moreover, in the case of cancer-related fatigue, the field has recognized the importance of this construct and it satisfies the definition of a proximal symptomatic measure of cancer severity [22].

The ECOG performance status [18], a clinical measure of disease severity, was also used as a known-groups validator for this psychometric analysis. The ECOG criteria are used to assess how a patient's disease is progressing and the effect of the disease on a patient’s activities of daily living and was assessed at the baseline visit.

In addition, demographic and medical history data, including age, sex, race, geographic region, line of therapy, and viral hepatitis infection status, were collected at the screening visit.

### Statistical analyses

In accordance with existing and emerging FDA guidance [13, 21], psychometric validation of the QLQ-HCC18 was conducted to measure the reliability (internal consistency and test–retest), construct validity (convergent and discriminant validity and known-groups validity), ability to detect change, and MWPC. These analyses were conducted using the safety population, which included all patients receiving at least one dose of tislelizumab. Known-groups validity and MWPC analyses were stratified on several pre-defined subpopulations, including region (China/Taiwan versus Europe), line of therapy (second-line versus third-line or greater), and viral hepatitis infection status (HBV/HCV positive versus hepatitis negative). Table 1 provides a summary of these analyses.

**Table 1:** Summary of psychometric analyses of QLQ-HCC18

Property	Analysis period	Definition	Test	Success criterion
Internal consistency	Baseline	Cronbach’s α	No test, point estimate reported	0.70 ≤ α
Test–retest reliability	Baseline to week 3	ICC(2,1)	No test, point estimate reported	0.70 ≤ ICC(2,1)
Concurrent validity	Baseline	Spearman correlations	No test, point estimate reported	|r|≥ 0.4
Known-groups validity	Baseline	Mean, mean difference, 95% CI, *P*-value, R^2^ effect size	ANOVA	*P* < 0.05; effect size ≥ 5%
Ability to detect change	Baseline to week 9	Mean change from baseline in scores between anchor (QLQ-C30 GHS), 95% CI, *P*-value, and ω^2^ semi-partial effect size	ANCOVA	*P* < 0.05; effect size ≥ 5%
Meaningful within-patient change	Baseline to week 9	Mean change from baseline in relation to change in anchor groups (QLQ-C30 GHS improvement, maintenance, deterioration) eCDFs plotted	No test, point estimates reported	No criterion, estimates reported

*ANCOVA* analysis of covariance, *ANOVA* analysis of variance, *CI* confidence interval, *eCDF* empirical cumulative distribution function, *ICC* intraclass correlation coefficient, *QLQ-C30 GHS* Quality of Life Questionnaire – Core 30 global health status/QoL scale, *QLQ-HCC18* Quality of Life Questionnaire – Hepatocellular Carcinoma 18-question module

Descriptive statistics for continuous variables were reported as means, standard deviations (SDs), medians, and missing values. Descriptive statistics for categorical variables were reported as frequency counts and the percentage of patients in corresponding categories. Statistical significance was evaluated using a two-tailed α = 0.05 level. Missing data for the QLQ-HCC18 and QLQ-C30 were handled according to the developer’s manuals and no imputation was carried out [21, 23]. All analyses were performed using SAS (version 9.4) and R statistical software (version 3.6.1).

It is important to note that several analyses were stratified by region (strata: China/Taiwan and Europe). These included known-groups validity and meaningful within-patient change. This stratification was motivated by guidance from the Chinese National Medical Products Administration (NMPA), which requires stratification to demonstrate the evidence unique to the Chinese population and whether this differs from the aggregate findings.

#### Reliability

Internal consistency evaluates score reliability by assessing the strength with which each item measures an assumed single domain. Internal consistency was assessed for each of the multi-item QLQ-HCC18 scales at baseline using Cronbach’s alpha [24]. Internal consistency estimates of ≥ 0.70 were considered acceptable [19].

Test–retest reliability consists of measuring the degree to which an instrument is capable of reproducing scores across time in patients whose condition has not changed [21]. Patients whose responses on the QLQ-C30 GHS scale anchor reflected no change in status between baseline and the first follow-up at week 3 were considered a stable subgroup and test–retest reliability was assessed for each of the QLQ-HCC18 scales and single items. In the case of a continuous score, one appropriate measure of test–retest reliability is the two-way random intraclass correlation coefficient (ICC), employed in this analysis and denoted as ICC(2,1) [25]. Test–retest reliability estimates of ≥ 0.70 indicate satisfactory reliability [26]. Both unconditional estimates and estimates conditioned on no change in GHS were applied. Consistent with regulatory guidance, only estimates derived from the primary GHS anchor-based no-change definition (NC1, defined by GHS change score of 0 between baseline and week 3) are reported [13, 21, 27].To limit the impact of possible treatment effects, three definitions of no change were examined in sensitivity analyses: unconditional, + 1 response category (‘NC2’), or + 2 response categories (‘NC3’). None of these definitions outperformed the pre-specified primary NC1 definition reported in this manuscript.

#### Construct validity

Construct validity was assessed by tests of both convergent and discriminant validity and known-groups validity. Convergent and discriminant validity is a component of construct validity representing the extent to which two scales assessing similar constructs are related. This was estimated from Spearman correlations between the QLQ-HCC18 and QLQ-C30 scores at baseline. Moderate to strong correlations reflect convergent validity while small correlations reflect discriminant validity [27]. Correlations between QLQ-HCC18 domains (which are symptom-focused) were expected to correlate positively with QLQ-C30 symptom domains, negatively with QLQ-C30 functional domains, and negatively with QLQ-C30 GH domains. For example, the QLQ-HCC18 fatigue domain was expected to correlate with the QLQ-C30 fatigue domain strongly and positively. The QLQ-HCC18 fatigue domain was expected to correlate with the QLQ-C30 physical function domain moderately and negatively. Finally, the QLQ-HCC18 fatigue domain was expected to correlate with the QLQ-C30 GHS moderately and negatively. Spearman correlations of |r|≥ 0.40 met the pre-specified criterion for acceptable convergent validity [26]. Given the exploratory nature of this analysis within this population for the purposes of identification of relevant phase 3 endpoints, no further hypotheses were specified for correlation-based analyses.

Known-groups validity assesses whether PRO scores can be differentiated between clinically distinct groups. Known-groups validity was estimated for the QLQ-HCC18 scores at baseline. Known-groups validators included geographic region (Asia versus Europe), line of therapy (second-line versus third-line or greater), ECOG status (0 versus 1), and viral hepatitis infection status (HBV/HCV positive versus hepatitis negative). Consistent with previous studies, the hypothesized direction of effect predicted that Europe would report lower quality of life than Asia [12, 28], third-line or greater would report lower QoL than second line, worse ECOG status would report lower QoL than better ECOG status, and that HBV/HCV infected patients would report lower quality of life than non-infected patients. The difference in QLQ-HCC18 scores between each known-group was calculated and contrasted using analysis of variance (ANOVA), from which the mean difference between known-groups, corresponding 95% confidence interval (CI), *P*-value, and R-squared (R^2^) effect size were estimated. Acceptable known-groups validity was achieved if a preponderance of the known-effect-groups had QLQ-HCC18 mean scores consistent with clinical expectations (i.e., more severe groups had worse symptoms or HRQoL compared to less severe groups). Such evidence was strengthened if and when the corresponding differences across known-groups were statistically significant and the corresponding R^2^ was greater than 5%. Methods to correct for multiple comparisons were not employed as part of the known-groups analysis.

### Ability to detect change

Ability to detect change is a facet of longitudinal validity that evaluates the relationship between changes in the PRO instrument of interest over time in the context of changes in external criteria (i.e., ‘anchors’) [29]. Ability to detect change was assessed by analyzing the extent to which QLQ-HCC18 change scores could be predicted by change in the QLQ-C30 GHS anchor variable. The QLQ-C30 GHS anchor groups were operationalized as follows: improvement was defined by > 0-point change from baseline to week 9; maintenance was defined as 0-point change from baseline to week 9; deterioration was defined as < 0-point change from baseline to week 9.

Analysis of covariance (ANCOVA) was used to estimate differences in QLQ-HCC18 change score marginal means across QLQ-C30 GHS anchor groups (improvement [effect] versus maintenance [reference]; deterioration [effect] versus maintenance [reference]), controlling for age, sex, region, and baseline QLQ-HCC18 mean. Effect size estimates were based on the Omega squared (ω^2^) statistic [30].^1^ Acceptable ability to detect change was pre-specified as estimates meeting the following criteria: significant differences *(P* < 0.05) in marginal means across anchor group contrasts and effect sizes exceeding 5%.

### Meaningful within-patient change

Traditional estimation of meaningful change thresholds has relied on distribution and anchor-based methods. Increasingly, regulatory reviewers are emphasizing the latter; therefore, anchor-based methods were the focus of the current analyses [13, 21, 24]. Furthermore, such estimates have emphasized between-group differences (e.g., minimally important differences or minimal clinically important differences). The FDA has justifiably taken the position that within-patient change is not acceptably approximated from between-group differences. Instead, regulatory guidance emphasizes MWPC for the derivation of clinical significance estimates [21].

Anchor-based methods aim to define the magnitude of MWPC on a PRO instrument of interest among patients classified as experiencing meaningful change (improvement/deterioration) on an ‘anchor.’ Anchor-based MWPC thresholds were obtained via calculation of mean change in QLQ-HCC18 scores from baseline to week 9 stratified on the QLQ-C30 GHS anchor groups described above. In addition to primary analyses based on the total sample, meaningful improvement estimates were stratified by geographic region (Asia versus Europe), line of therapy (second-line versus third-line or greater), and viral hepatitis infection status (HBV/HCV positive versus hepatitis negative). These stratified estimates were employed to assess the uniformity in clinical significance threshold estimates across known subgroups within the trial, and to characterize unique effects within the China/Taiwan population, as required by NMPA guidance. These estimates of mean change were then validated by visualizing differences in cumulative proportions achieving the point estimates stratified on anchor groups via empirical cumulative distribution functions (eCDFs) and empirical probability density functions (ePDFs).

## Results

A total of 249 patients (138 s-line and 111 third-line or greater) were enrolled from 45 international centers in the BGB-A317-208 trial. A sample size of 228 was calculated to provide a power of 0.97 to demonstrate that the objective response rate in patients with previously treated unresectable HCC is statistically higher than the historical rate of 7% in a binomial exact test at a one-sided alpha level of 0.025. The demographics and clinical characteristics of these patients are summarized in Table 2. Patients had an average age of 60.3 years, were mostly male (87.1%), 50.6% were Asian, approximately half had an ECOG score of 1, and the average elapsed time from diagnosis to first dose of study drug was 38.7 months. Approximately one third of the patients (36.1%) were not HBV/HCV infected and approximately half were experiencing progressive disease prior to entering the study (51.4%). The average elapsed time from last systemic therapy dose to first study dose was 3.4 months. These patterns were similar across second-line and third-line or greater cohorts. A single patient who did not contribute QLQ-HCC18 data at baseline was excluded, leaving a final sample of 248 patients for the psychometric analyses.

**Table 2:** Patient demographics and clinical characteristics

Characteristic	Total sample (N = 249)^a^	Line of therapy	
		Second-line (n = 138)	Third-line or Greater(n = 111)
Age (years)			
Mean (SD)	60.3 (12.5)	60.2 (13.7)	60.4 (10.9)
Median	62.0	63.5	60.0
Min, Max	28, 90	28, 90	28, 82
Age group, n (%)			
< 65 years	149 (59.8)	75 (54.3)	74 (66.7)
≥ 65 years	100 (40.2)	63 (45.7)	37 (33.3)
Sex, *n* (%)			
Male	217 (87.1)	121 (87.7)	96 (86.5)
Female	32 (12.9)	17 (12.3)	15 (13.5)
Race, *n* (%)			
Asian	126 (50.6)	74 (53.6)	52 (46.8)
Black or African American	4 (1.6)	2 (1.4)	2 (1.8)
White	96 (38.6)	43 (31.2)	53 (47.7)
Other	2 (0.8)	2 (1.4)	0 (0.0)
Not reported	21 (8.4)	17 (12.3)	4 (3.6)
ECOG performance status at baseline, *n* (%)			
0	129 (51.8)	70 (50.7)	59 (53.2)
1	120 (48.2)	68 (49.3)	52 (46.8)
Time from initial diagnosis to the first study dose (months)			
N	249	138	111
Mean (SD)	38.7 (39.6)	35.7 (37.8)	42.5 (41.6)
Median	24.9	21.4	28.1
Min, Max	0.3, 269.6	2.3, 267.1	0.3, 269.6
Child–Pugh classification at baseline, n (%)			
A	248 (99.6)	138 (100.0)	110 (99.1)
B	1 (0.4)	0 (0.0)	1 (0.9)
Alpha-fetoprotein at baseline (ng/ml)			
> 200 ng/mL	128 (51.4)	62 (44.9)	66 (59.5)
> 400 ng/mL	112 (45.0)	53 (38.4)	59 (53.2)
Hepatitis virus infection, *n* (%)			
Uninfected	90 (36.1)	46 (33.3)	44 (39.6)
Hepatitis B only	123 (49.4)	71 (51.4)	52 (46.8)
Hepatitis C only	31 (12.4)	20 (14.5)	11 (9.9)
Coinfected	5 (2.0)	1 (0.7)	4 (3.6)
Number of lines of prior systemic therapy received, *n* (%)			
0	1 (0.4)	1 (0.7)	0 (0.0)
1	137 (55.0)	137 (99.3)	0 (0.0)
2	102 (41.0)	0 (0.0)	102 (91.9)
≥ 3	9 (3.6)	0 (0.0)	9 (8.1)
Best response to last systemic therapy, n (%)			
Complete response	0 (0.0)	0 (0.0)	0 (0.0)
Partial response	11 (4.4)	6 (4.3)	5 (4.5)
Stable disease	68 (27.3)	36 (26.1)	32 (28.8)
Progressive disease	128 (51.4)	71 (51.4)	57 (51.4)
Unknown/Not applicable/Missing	42 (16.9)	25 (18.1)	17 (15.3)
Time from the end of last systematic therapy to first dose (months)			
Mean (SD)	3.4 (6.8)	4.0 (8.6)	2.5 (3.0)
Median	1.4	1.5	1.4
Min, Max	0.5, 79.0	0.5, 79.0	0.5, 17.7

*ECOG* Eastern Cooperative Oncology Group, *QLQ-HCC18*, Quality of Life Questionnaire – Hepatocellular Carcinoma 18-question module, *SD* standard deviation

^a^A single patient who did not contribute QLQ-HCC18 data at baseline was excluded, leaving a final sample of 248 patients for the psychometric analyses

### Reliability

The Cronbach’s alpha coefficients of three QLQ-HCC18 domains, namely fatigue, nutrition, and index reflected acceptable internal consistency (0.71, 0.75, and 0.88, respectively). The remaining multi-item domains of body image, jaundice, pain, and fever did not display satisfactory internal consistency for this patient population (< 0.70).

Within the two assessments (baseline and 3-week follow-up) and across domains, 85–87 patients were included within the primary GHS-based no-change (NC1) population upon which test–retest reliability was estimated. Test–retest reliability ICC(2,1) estimates indicated satisfactory reliability for six QLQ-HCC18 domains: fatigue, body image, nutrition, pain, sexual interest, and index (0.72, 0.70, 0.73, 0.75, 0.79, and 0.83 respectively). The remaining domains of jaundice, fever, and abdominal swelling did not display adequate test–retest reliability (< 0.70).

### Construct validity

Convergent and discriminant validity estimates are presented in Table 3. Results were largely consistent with expectations for which QLQ-HCC18 domains would demonstrate a preponderance of acceptable associations. Correlations between QLQ-HCC18 scores and QLQ-C30 fatigue, nausea and vomiting, and pain domains met or exceeded the pre-specified criterion of |r|≥ 0.4. As expected, the QLQ-HCC18 fatigue and pain domains correlated with QLQ-C30 fatigue and pain domains strongly and positively (0.76 and 0.60). The HCC18 fatigue domain correlated with QLQ-C30 physical function and GHS strongly and negatively (–0.7 and –0.51–0.52). The fatigue domain achieved this pre-specified criterion for 13 out of 16 (81.3%) correlations, whereas the index score achieved this pre-specified criterion for 14 out of 16 (87.5%) correlations. Conversely, there were weak correlations between domains and items assessing divergent concepts, suggesting acceptable discriminant validity. For example, the correlation between the QLQ-HCC18 fever domain and the QLQ-C30 financial difficulties item was 0.21. The jaundice domain did not achieve the pre-specified criterion for any of the 16 correlations.

**Table 3:** Convergent and discriminant validity for the QLQ-HCC18 domains and the QLQ-C30 scores at baseline

QLQ-C30 Validators	QLQ-HCC18 Domains								
	Abdominal Swelling	Body Image	Fever	Fatigue	Jaundice	Nutrition	Pain	Sexual Interest	Index
Physical functioning	− 0.46	− 0.59	− 0.36	− 0.70	− 0.27	− 0.56	− 0.56	− 0.34	− 0.71
Role functioning	− 0.32	− 0.55	− 0.36	− 0.62	− 0.29	− 0.51	− 0.45	− 0.35	− 0.63
Emotional functioning	− 0.40	− 0.58	− 0.41	− 0.59	− 0.33	− 0.47	− 0.53	− 0.29	− 0.64
Cognitive functioning	− 0.22	− 0.49	− 0.43	− 0.55	− 0.30	− 0.37	− 0.44	− 0.30	− 0.56
Social functioning	− 0.26	− 0.44	− 0.39	− 0.55	− 0.18	− 0.51	− 0.32	− 0.46	− 0.59
Fatigue	0.41	0.60	0.39	0.76	0.29	0.56	0.55	0.34	0.71
Nausea and vomiting	0.37	0.52	0.43	0.39	0.31	0.56	0.49	0.26	0.59
Pain	0.39	0.50	0.36	0.60	0.29	0.48	**0.60**	0.29	0.63
Dyspnea	0.34	0.54	0.46	0.48	0.32	0.41	0.36	0.34	0.59
Insomnia	0.19	0.34	0.29	0.38	0.22	0.26	0.36	0.21	0.40
Appetite	0.29	0.50	0.35	0.49	0.31	0.68	0.44	0.20	0.57
Constipation	0.25	0.28	0.21	0.33	0.20	0.39	0.31	0.12	0.36
Diarrhea	0.36	0.44	0.26	0.30	0.18	0.33	0.34	0.12	0.41
Financial difficulties	0.16	0.18	0.21	0.24	− 0.02	0.23	0.15	0.38	0.32
GHS1	− 0.33	− 0.49	− 0.34	− 0.52	− 0.22	− 0.45	− 0.44	− 0.30	− 0.56
GHS2	− 0.34	− 0.49	− 0.31	− 0.51	− 0.19	− 0.44	− 0.41	− 0.33	− 0.56

*GHS* Global health status/QoL scale, *QLQ-C30* Quality of Life Questionnaire – Core 30, *QLQ-HCC18* Quality of Life Questionnaire – Hepatocellular Carcinoma 18-question module

The known-groups validity estimates are presented in Table 4. Known-groups validity of QLQ-HCC18 domains at baseline was defined upon geographic region, line of therapy, ECOG status, and viral hepatitis status. As hypothesized, European patients had a significantly higher mean score for domains of fatigue (group difference: 5.28, *P* = 0.025), body image (group difference: 8.32, *P* < 0.001), jaundice (group difference: 4.88, *P* = 0.001), pain (group difference: 5.73, *P* = 0.010), and index (group difference: 4.85, *P* = 0.002) compared with Asian patients, respectively. These mean differences were associated with effect sizes (R^2^) indicating 2% to 5% explained variance. A non-significant trend was observed for the remaining domains/items, whereby European patients had higher mean scores. As expected, patients in the third-line or greater therapy group had higher mean scores for all domains/items compared with patients in the second-line therapy group; however, only the jaundice domain demonstrated a significant group difference (group difference: 4.50, *P* = 0.003).

**Table 4:** Known-groups validity for QLQ-HCC18 domain and item scores at baseline

Validator	Contrast	N	Group means	Group difference	95% CI	*P*-value	Effect size (R^2^)
*Fatigue domain*							
Geographic region	China/Taiwan (Ref) Europe	122 126	19.76 25.04	5.28	0.67, 9.89	0.025	0.02
Treatment line	2nd line (Ref) ≥ 3rd line	137 111	21.98 23.02	1.04	− 3.64, 5.73	0.661	0.00
Baseline ECOG score	0 (Ref) 1	131 117	20.61 24.50	3.89	− 0.75, 8.53	0.100	0.01
Baseline viral hepatitis status	Hepatitis + (Ref) Hepatitis −	158 90	21.52 24.07	2.56	− 2.28, 7.39	0.299	0.00
*Body image domain*							
Geographic region	China/Taiwan (Ref) Europe	122 126	8.61 16.93	8.32	3.78, 12.87	< 0.001	0.05
Treatment line	2nd line (Ref) ≥ 3rd line	137 111	12.17 13.66	1.50	− 3.19, 6.18	0.529	0.00
Baseline ECOG score	0 (Ref) 1	131 117	10.94 14.96	4.02	− 0.63, 8.66	0.090	0.01
Baseline viral hepatitis status	Hepatitis + (Ref) Hepatitis −	158 90	10.76 16.48	5.72	0.93, 10.52	0.020	0.02
*Jaundice domain*							
Geographic region	China/Taiwan (Ref) Europe	122 126	4.64 9.52	4.88	1.90, 7.86	0.001	0.04
Treatment line	2nd line (Ref) ≥ 3rd line	137 111	5.11 9.61	4.50	1.50, 7.50	0.003	0.03
Baseline ECOG score	0 (Ref) 1	131 117	7.38 6.84	− 0.54	− 3.58, 2.50	0.726	0.00
Baseline viral hepatitis status	Hepatitis + (Ref) Hepatitis −	158 90	6.33 8.52	2.19	− 0.96, 5.34	0.172	0.01
*Nutrition domain*							
Geographic region	China/Taiwan (Ref) Europe	122 126	9.95 12.86	2.91	− 0.82, 6.65	0.126	0.01
Treatment line	2nd line (Ref) ≥ 3rd line	137 111	10.95 12.01	1.06	− 2.71, 4.83	0.579	0.00
Baseline ECOG score	0 (Ref) 1	131 117	10.13 12.88	2.75	− 0.99, 6.49	0.149	0.01
Baseline viral hepatitis status	Hepatitis + (Ref) Hepatitis −	158 90	11.60 11.11	− 0.49	− 4.39, 3.41	0.804	0.00
*Pain domain*							
Geographic region	China/Taiwan (Ref) Europe	122 126	11.20 16.93	5.73	1.39, 10.07	0.010	0.03
Treatment line	2nd line (Ref) ≥ 3rd line	137 111	13.63 14.71	1.09	− 3.33, 5.51	0.628	0.00
Baseline ECOG score	0 (Ref) 1	131 117	11.83 16.67	4.83	0.47, 9.20	0.030	0.02
Baseline viral hepatitis status	Hepatitis + (Ref) Hepatitis −	158 90	12.66 16.67	4.01	− 0.54, 8.56	0.084	0.01
*Fever domain*							
Geographic region	China/Taiwan (Ref) Europe	122 126	4.10 4.89	0.80	− 1.65, 3.24	0.522	0.00
Treatment line	2^nd^ line (Ref) ≥ 3^rd^ line	137 111	3.65 5.56	1.91	− 0.55, 4.36	0.127	0.01
Baseline ECOG score	0 (Ref) 1	131 117	5.60 3.28	− 2.32	− 4.76, 0.11	0.062	0.01
Baseline viral hepatitis status	Hepatitis + (Ref) Hepatitis −	158 90	4.75 4.07	− 0.67	− 3.22, 1.87	0.603	0.00
*Abdominal swelling item*							
Geographic region	China/Taiwan (Ref) Europe	122 125	12.30 16.27	3.97	− 1.36, 9.30	0.143	0.01
Treatment line	2^nd^ line (Ref) ≥ 3^rd^ line	137 110	12.90 16.06	3.17	− 2.20, 8.53	0.247	0.01
Baseline ECOG score	0 (Ref) 1	131 116	13.23 15.52	2.29	− 3.07, 7.64	0.401	0.00
Baseline viral hepatitis status	Hepatitis + (Ref) Hepatitis −	158 89	14.77 13.48	− 1.28	− 6.86, 4.29	0.650	0.00
*Sex life item*							
Geographic region	China/Taiwan (Ref) Europe	121 123	21.21 29.00	7.79	− 0.41, 15.98	0.063	0.01
Treatment line	2nd line (Ref) ≥ 3rd line	135 109	23.21 27.52	4.31	− 3.97, 12.60	0.306	0.00
Baseline ECOG score	0 (Ref) 1	129 115	24.03 26.38	2.35	− 5.92, 10.61	0.576	0.00
Baseline viral hepatitis status	Hepatitis + (Ref) Hepatitis −	157 87	26.96 21.84	− 5.12	− 13.72, 3.47	0.241	0.01
*Index*							
Geographic region	China/Taiwan (Ref) Europe	122 127	11.46 16.31	4.85	1.84, 7.86	0.002	0.04
Treatment line	2nd line (Ref) ≥ 3rd line	138 111	12.91 15.18	2.27	− 0.81, 5.35	0.148	0.01
Baseline ECOG score	0 (Ref) 1	131 118	12.95 15.02	2.06	− 1.01, 5.13	0.187	0.01
Baseline viral hepatitis status	Hepatitis + (Ref) Hepatitis −	159 90	13.59 14.52	0.94	− 2.26, 4.13	0.564	0.00

*CI* confidence interval, *ECOG* Eastern Cooperative Oncology Group, *QLQ-HCC18* Quality of Life Questionnaire – Hepatocellular Carcinoma 18-question module

Patients with an ECOG score of 1 had a significantly higher mean score for the pain domain (group difference: 4.83, *P* = 0.030) compared with patients that had an ECOG score of 0. An unexpected trend was observed for jaundice (group difference: − 0.54, P = 0.726) and fever (group difference: − 2.32, *P* = 0.062), whereby patients with an ECOG score of 0 had higher scores. As expected, patients in the HBV/HCV positive group had higher mean scores for domains of nutrition (group difference: − 0.49, *P* = 0.804), fever (group difference: − 0.67, *P* = 0.603), abdominal swelling (group difference: − 1.28, *P* = 0.650), and sex life (group difference: – 5.12, *P* = 0.241). An unexpected trend was observed for fatigue, body image, jaundice, pain, and index, whereby patients in the HBV/HCV-negative group had higher mean scores.

The majority of known-groups validity estimates (81%) were consistent with the hypothesized direction of effect, thereby supporting validity of the QLQ-HCC18.

### Ability to detect change

Change scores were computed for the QLQ-HCC18 scores based on the QLQ-C30 GHS scale anchor groups of improvement, maintenance, and deterioration. The ability to detect change estimates are presented in Table 5. Clear differentiation of the QLQ-HCC18 change scores between improvement and maintenance groups were observed for body image, fatigue, pain, and index. Effect sizes were small (less than 0.10), most likely induced by the large variability in these data relative to the reasonable sample sizes, as indicated by the wide 95% CIs. No statistically significant changes were observed between improvement and maintenance groups for abdominal swelling, fever, jaundice, nutrition, and sexual interest. Clear differentiation of QLQ-HCC18 change scores between deterioration and maintenance groups were observed for fever and fatigue. No statistically significant differentiation was observed for the remaining QLQ-HCC18 symptom scores, including index.

**Table 5:** QLQ-HCC18 ability to detect change scores from baseline to week 9 by anchor group

QLQ-HCC18 domain^a^	QLQ-C30 GHS anchor^b^	Group Difference^c^	95% CI	*P*-value	Total omega effect size	QLQ omega effect size
Abdominal swelling	Improve (n = 50) vs. Maintenance (n = 63)	− 5.98	− 12.91, 0.95	0.090	0.036	0.017
	Deteriorate (n = 61) vs. Maintenance (n = 63)	2.39	− 4.09, 8.87	0.466	− 0.008	− 0.004
Body image	Improve (n = 47) vs. Maintenance (n = 63)	− 10.26	− 16.55, − 3.96	0.002	0.047	0.083
	Deteriorate (n = 63) vs. Maintenance (n = 63)	0.40	− 4.03, 4.83	0.859	0.028	− 0.008
Fever	Improve (n = 49) vs. Maintenance (n = 62)	− 1.28	− 4.75, 2.19	0.467	− 0.006	− 0.004
	Deteriorate (n = 63) vs. Maintenance (n = 62)	7.23	3.2, 11.25	0.001	0.002	0.094
Fatigue	Improve (n = 50) vs. Maintenance (n = 63)	− 6.59	− 12.65, − 0.53	0.033	0.026	0.032
	Deteriorate (n = 61) vs. Maintenance (n = 63)	6.34	0.97, 11.72	0.021	− 0.005	0.036
Jaundice	Improve (n = 50) vs. Maintenance (n = 63)	− 2.90	− 7.21, 1.4	0.184	0.015	0.007
	Deteriorate (n = 62) vs. Maintenance (n = 63)	− 0.33	− 4.48, 3.82	0.876	0.001	− 0.008
Nutrition	Improve (n = 49) vs. Maintenance (n = 62)	− 4.32	− 9.07, 0.43	0.075	0.008	0.020
	Deteriorate (n = 61) vs. Maintenance (n = 62)	3.23	− 0.36, 6.83	0.078	− 0.008	0.018
Pain	Improve (n = 49) vs. Maintenance (n = 61)	− 5.44	− 10.73, − 0.16	0.044	0.056	0.027
	Deteriorate (n = 63) vs. Maintenance (n = 61)	− 0.14	− 5.44, 5.16	0.958	− 0.008	− 0.008
Sexual interest	Improve (n = 49) vs. Maintenance (n = 62)	− 4.18	− 13.89, 5.54	0.396	− 0.007	− 0.003
	Deteriorate (n = 60) vs. Maintenance (n = 62)	− 1.14	− 10.43, 8.16	0.809	− 0.007	− 0.008
Index	Improve (n = 50) vs. Maintenance (n = 64)	− 5.31	− 8.56, − 2.05	0.002	0.071	0.078
	Deteriorate (n = 63) vs. Maintenance (n = 64)	2.23	− 0.63, 5.09	0.125	− 0.007	0.011

*CI* confidence interval, *QLQ-30 GHS* Quality of Life Questionnaire Cancer – Core 30 global health status/QoL scale, *QLQ-HCC18* Quality of Life Questionnaire – Hepatocellular Carcinoma 18-question module

^a^QLQ-HCC18 domains are scored on a scale of 0–100 with higher scores indicate worse symptoms or more problems

^b^QLQ-C30 GHS is scored on a scale of 1 to 7 with lower scores indicating reduced or low quality of life. Improve was defined as > 0-point change in QLQ-C30 GHS score; maintenance was defined as 0-point change; deterioration was defined as < 0-point change

^c^Difference in marginal mean change score between anchors

### Meaningful within-patient change

The point estimates for MWPC across anchor groups are presented for the total sample and stratified by region, line of therapy, and viral hepatitis infection status in Table 6. Within the primary (unstratified) analyses, point estimates for MWPC defining improvement were –7.18 for QLQ-HCC18 fatigue and − 4.07 for QLQ-HCC18 index. Meaningful improvement estimates for the index scale stratified on either region or HBV/HCV infection were identical to the primary estimates. Region-stratified estimates of meaningful improvement for fatigue were within ± 1 point of the primary estimates. Line of therapy stratified estimates were within ± 2 of primary estimates for both fatigue and index. The viral hepatitis negative sample achieved greater fatigue improvement (− 10) compared to the HBV/HCV infected sample (− 5).

**Table 6:** QLQ-HCC18 meaningful within-patient change estimates from baseline to week 9 by anchor group

QLQ-HCC18 domain^a^	QLQ-C30 GHS anchor^b^	Mean change						
		Total sample	China/Taiwan	Europe	Second-line therapy	Third-line or greater therapy	Viral hepatitis negative	HBV/HCV positive
Abdominal swelling	Deteriorate Improve Maintenance	4.97 (n = 64)	6 (n = 32)	4 (n = 32)	3 (n = 30)	6 (n = 34)	8 (n = 24)	3 (n = 40)
		− 2.66 (n = 50)	− 5 (n = 19)	− 1 (n = 31)	− 3 (n = 30)	− 2 (n = 20)	3 (n = 22)	− 7 (n = 28)
		2.65 (n = 64)	− 1 (n = 31)	6 (n = 33)	3 (n = 34)	2 (n = 30)	6 (n = 24)	1 (n = 40)
Body image	Deteriorate Improve Maintenance	2.92 (n = 64)	− 2 (n = 32)	8 (n = 32)	− 1 (n = 30)	6 (n = 34)	6 (n = 24)	1 (n = 40)
		− 7.49 (n = 50)	− 5 (n = 19)	− 9 (n = 31)	− 7 (n = 30)	− 8 (n = 20)	− 13 (n = 22)	− 4 (n = 28)
		2.63 (n = 64)	0 (n = 31)	5 (n = 33)	3 (n = 34)	2 (n = 30)	6 (n = 24)	1 (n = 40)
Fever	Deteriorate Improve Maintenance	6.06 (n = 64)	4 (n = 32)	9 (n = 32)	4 (n = 30)	8 (n = 34)	9 (n = 24)	4 (n = 40)
		− 2.39 (n = 50)	0 (n = 19)	− 4 (n = 31)	− 3 (n = 30)	− 1 (n = 20)	− 5 (n = 22)	− 1 (n = 28)
		− 1.13 (n = 64)	− 3 (n = 31)	1 (n = 33)	− 1 (n = 34)	− 2 (n = 30)	4 (n = 24)	− 4 (n = 40)
Fatigue	Deteriorate Improve Maintenance	5.34 (n = 64)	5 (n = 32)	6 (n = 32)	2 (n = 30)	9 (n = 34)	5 (n = 24)	5 (n = 40)
		− 7.18 (n = 50)	− 6 (n = 19)	− 8 (n = 31)	− 9 (n = 30)	− 5 (n = 20)	− 10 (n = 22)	− 5 (n = 28)
		− 0.87 (n = 64)	− 4 (n = 31)	2 (n = 33)	0 (n = 34)	− 2 (n = 30)	2 (n = 24)	− 2 (n = 40)
Jaundice	Deteriorate Improve Maintenance	2.18 (n = 64)	2 (n = 32)	2 (n = 32)	2 (n = 30)	2 (n = 34)	2 (n = 24)	2 (n = 40)
		− 0.06 (n = 50)	1 (n = 19)	− 1 (n = 31)	− 2 (n = 30)	2 (n = 20)	0 (n = 22)	0 (n = 28)
		2.57 (n = 64)	0 (n = 31)	5 (n = 33)	4 (n = 34)	0 (n = 30)	8 (n = 24)	− 1 (n = 40)
Nutrition	Deteriorate Improve Maintenance	2.72 (n = 64)	1 (n = 32)	4 (n = 32)	2 (n = 30)	4 (n = 34)	3 (n = 24)	3 (n = 40)
		− 4.67 (n = 50)	− 3 (n = 19)	− 6 (n = 31)	− 7 (n = 30)	− 1 (n = 20)	− 6 (n = 22)	− 3 (n = 28)
		− 0.44 (n = 64)	− 2 (n = 31)	1 (n = 33)	0 (n = 34)	− 1 (n = 30)	2 (n = 24)	− 2 (n = 40)
Pain	Deteriorate Improve Maintenance	2.3 (n = 64)	6 (n = 32)	− 2 (n = 32)	2 (n = 30)	2 (n = 34)	− 2 (n = 24)	5 (n = 40)
		− 2.35 (n = 30)	− 5 (n = 19)	− 1 (n = 31)	− 6 (n = 30)	3 (n = 20)	− 1 (n = 22)	− 4 (n = 28)
		2.44 (n = 64)	− 1 (n = 31)	6 (n = 33)	5 (n = 34)	− 1 (n = 30)	4 (n = 24)	1 (n = 40)
Sexual interest	Deteriorate Improve Maintenance	− 1.73 (n = 64)	− 3 (n = 32)	0 (n = 32)	5 (n = 30)	− 7 (n = 34)	0 (n = 24)	− 3 (n = 40)
		− 4.78 (n = 50)	− 5 (n = 19)	− 4 (n = 31)	− 3 (n = 30)	− 7 (n = 20)	− 5 (n = 22)	− 5 (n = 28)
		− 0.56 (n = 64)	0 (n = 31)	− 1 (n = 33)	0 (n = 34)	− 1 (n = 30)	− 2 (n = 24)	0 (n = 40)
Index	Deteriorate Improve Maintenance	3.16 (n = 64)	2 (n = 32)	4 (n = 32)	2 (n = 30)	4 (n = 34)	4 (n = 24)	2 (n = 40)
		− 4.07 (n = 50)	− 4 (n = 19)	− 4 (n = 31)	− 5 (n = 30)	− 2 (n = 20)	− 5 (n = 22)	− 4 (n = 28)
		1 (n = 64)	− 1 (n = 31)	3 (n = 33)	2 (n = 34)	0 (n = 30)	4 (n = 24)	− 1 (n = 40)

*HBV/HCV* hepatitis B virus/hepatitis C virus, *QLQ-30 GHS* Quality of Life Questionnaire Cancer – Core 30 global health status/QoL scale, *QLQ-HCC18* Quality of Life Questionnaire – Hepatocellular Carcinoma 18-question module

^1^QLQ-HCC18 domains are scored on a scale of 0–100 with higher scores indicating worse symptoms or more problems

^2^QLQ-C30 GHS is scored on a scale of 1 to 7 with lower scores indicating reduced or low quality of life. Improve was defined as > 0-point change in QLQ-C30 GHS score; maintenance was defined as 0-point change; deterioration was defined as < 0-point change

Within the primary (unstratified) analyses, point estimates for MWPC defining deterioration for QLQ-HCC18 fatigue and index were 5.34 and 3.16, respectively. In the case of the fatigue domain, estimates stratifying on either region or HBV/HCV infection status were identical to the primary estimates (the one exception was Europe for which the estimate was 0.66 points higher). In the case of line of therapy, estimates were 2 and 9, respectively, for second-line and third-line or greater, reflecting greater heterogeneity relative to the primary estimates. In the case of the index scale, all stratified estimates were within ± 1 of the primary estimates and therefore unaltered across population stratification.

**Fig. 1:**
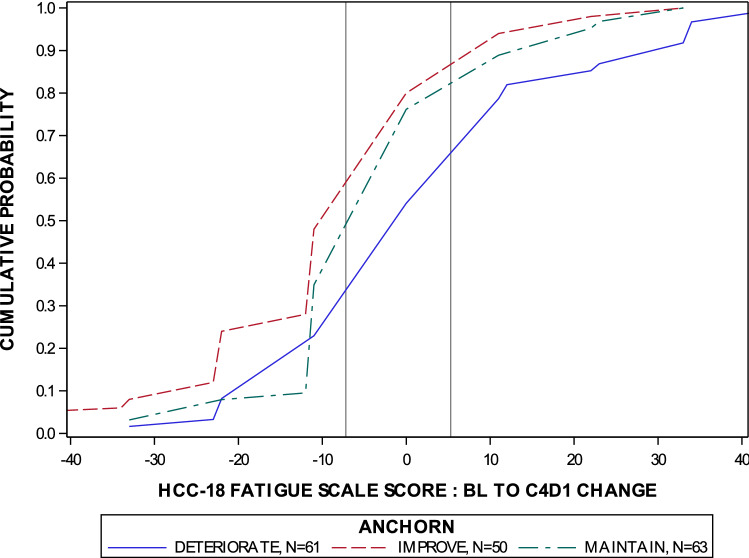
eCDF of QLQ-HCC18 fatigue domain change score from baseline to week 9 by anchor group. eCDF: empirical cumulative distribution function; *QLQ-HCC18* Quality of Life Questionnaire – Hepatocellular Carcinoma 18-question module

The point estimates for MWPC for each anchor group definition were validated by eCDF figure. In the case of meaningful improvement for fatigue domain scores, 60% of the improvement anchor group and 50% of the maintenance anchor group achieved the − 7.13 threshold, yielding a 10% improvement advantage. In the case of meaningful deterioration for fatigue scores, 38% of the deterioration anchor group and 18% of the maintenance anchor group achieved the 5.34 threshold, yielding a 20% advantage for maintenance. The eCDF for the QLQ-HCC18 fatigue score is presented in Fig. 1. The corresponding eCDF clarifies the overlap in fatigue domain change score distributions, but also demonstrates that the mass of distributions was offset as expected, with improvement skewed left, maintenance centered about a change score of zero, and deterioration skewed to the right.

## Discussion

The present study examined the psychometric properties, namely reliability, construct validity, ability to detect change, and MWPC, of the EORTC QLQ-HCC18 instrument within the BGB-A317-208 trial population of patients with unresectable HCC. Within this population, evidence suggested that the QLQ-HCC18 demonstrates heterogenous psychometric properties. However, the QLQ-HCC18 fatigue and index domains were found to consistently demonstrate robust psychometrics.

With respect to reliability, this study found that only the QLQ-HCC18 fatigue, nutrition, and index domains demonstrated acceptable internal consistency at baseline. This is not surprising given that previous validation studies found low alpha coefficients for the QLQ-HCC18 jaundice, pain, and fever domains, citing heterogeneity within the HCC patient population as the cause [7, 9, 12]. Specifically, these studies suggested heterogeneity of the items within the scales and within the patient population (e.g., region, viral hepatitis status) may be contributing factors. That may be the case, though a simpler explanation likely exists, and is reviewed within the limitations section. Acceptable test–retest reliability was found for fatigue, body image, nutrition, pain, sexual interest, and index. The observed low ICC estimates for the jaundice domain may have resulted from few patients presenting with jaundice upon admission to the trial.

Convergent and discriminant validity, as with all validation analyses within this phase 2 trial, were treated as exploratory and beyond hypotheses outlined in methods (i.e., direction of association with symptom, functional, and global health domains and the pre-specified criterion for acceptable association). No specific hypotheses for which domains would have greater or lesser association were pre-specified. Associations were exploratory and the preponderance of evidence examined to conclude broadly whether the associations with sufficient domains were detected to justify elevating a given QLQ-HCC18 domain from exploratory to secondary endpoint in a phase 3 clinical trial setting. Results were largely consistent with expectations for which QLQ-HCC18 domains would demonstrate a preponderance of acceptable associations. Going forward, this exploratory evidence will support confirmatory hypotheses in forthcoming phase 3 studies. The fatigue domain achieved this pre-specified criterion for 13 of the 16 correlations, whereas the index domain achieved this pre-specified criterion for 14 of the 16 correlations. This was true for both convergent and discriminant validators. Most of the correlations with the QLQ-HCC18 jaundice domain and sexual interest item failed to meet the pre-specified criterion; this finding is supported by previous validation studies that reported weak correlations between the QLQ-HCC18 jaundice and sexual interest and the QLQ-C30 scores, and FACT-Hepatobiliary scores [12, 13]. This is likely because these QLQ-HCC18 items are specific to symptoms/signs of HCC.

The majority of known-groups validity estimates (81%) were consistent with the hypothesized direction of effect, thereby supporting validity of the QLQ-HCC18. This suggests the QLQ-HCC18 can generally differentiate among distinct groups as hypothesized a priori*.*

Known-groups validity evidence for the geographic region effect was consistent with the hypothesized direction of effect, under which Europe was expected to report lower QoL/worse symptoms compared with Asia. This hypothesis was driven by findings reported by previous studies that demonstrate geographic areas effect HRQoL in HCC. Specifically, Asian patients with HCC report significantly better scores in HCC18 scales (sexual interest, fatigue) than European patients [31]. It has been posited that these differences in scores stem from variability in management practices between Europe and Asia (i.e., active surveillance programs implemented in Asia) [28].

Interpretable ability to detect change between patients improving versus maintaining according to the pre-specified QLQ-C30 GHS anchor thresholds was found for the fatigue, body image, pain, and index domain change scores. The same was found for ability to detect change between patients deteriorating versus maintaining for the fatigue domain. As expected, unbiased effect size estimates were low, indicating less than 10% explained variance across domains. This is often the case in oncology trials due to heterogeneity within the patient population, which increases dispersion, thereby attenuating effect-size magnitudes within the data.

To date, this is the first study to estimate MWPC thresholds in line with the methods outlined in the latest FDA guidance [21]. In this study, the estimated anchor-based MWPC threshold defining clinical significance for the fatigue domain was found to be lower than previously reported within the literature for the QLQ-C30 [32, 33]. This may be due to the difference between the minimally important difference and MWPC frameworks and has implications for the application of historical QLQ-C30 meaningful change thresholds outside of the original context of use. The revised MWPC deterioration estimates can be employed to define thresholds for progression endpoints, such as time to deterioration. The same is true for improvement endpoints, for which evidence was generated in this analysis indicating an ability of the QLQ-HCC18 fatigue domain to detect meaningful clinical improvement, which is a rare phenomenon in oncology PRO applications.

While the results of this study are important, they should be considered alongside some limitations. The most noteworthy limitation is that many of the QLQ-HCC18 domains did not consistently demonstrate optimal measurement properties in this HCC population. Specifically, body image, jaundice, pain, fever, and abdominal swelling did not display acceptable reliability. However, it is important to note that these domains consist of the fewest items within the QLQ-HCC18 instrument. Consistent with theory and previous evidence, the reliability of a score has been found to increase as the number of items contributing to the score increase [34, 35]. Additional limitations were related to validity and MWPC for domains other than fatigue and index. Jaundice and sexual interest failed to display acceptable validity. In addition, fever, nutrition, jaundice, abdominal swelling, and sexual interest did not show adequate ability to detect change.

Taken together, the validation evidence suggested that the QLQ-HCC18 fatigue and index domains consistently demonstrated robust psychometric properties. This appears to support the use of the fatigue and index domains as suitable patient-reported endpoints within an unresectable HCC population that had previously received one or more systemic therapies. Moreover, the ability to detect change and meaningful within-patient change analyses demonstrated that an uncommon degree of improvement was observed in this trial and the QLQ-HCC18 fatigue domain scores sensitively detected the effect of tislelizumab.

## Footnotes

1 The ${\omega }^{2}$ statistic is a less biased estimate, which corrects for sample size bias associated with other uncorrected effect size estimates such as Eta squared.

## References

[1] Hauser SL, Bar-Or A, Comi G, Giovannoni G, Hartung H-P, Hemmer B (2017). Ocrelizumab versus interferon beta-1a in relapsing multiple sclerosis. New England Journal of Medicine..

[2] El-Serag HB (2012). Epidemiology of viral hepatitis and hepatocellular carcinoma. Gastroenterology.

[3] Torre LA, Bray F, Siegel RL, Ferlay J, Lortet-Tieulent J, Jemal A (2015). Global cancer statistics 2012. A Cancer Journal for Clinicians.

[4] Ghouri Y, Mian I, Rowe J (2017). Review of hepatocellular carcinoma: Epidemiology, etiology, and carcinogenesis. Journal of Carcinogenesis..

[5] Bosch FX, Ribes J, Cléries R, Díaz M (2005). Epidemiology of hepatocellular carcinoma. Clinics in liver disease..

[6] Jemal A, Ward EM, Johnson CJ, Cronin KA, Ma J, Ryerson B (2017). Annual report to the nation on the status of cancer, 1975–2014 featuring survival. Journal of the National Cancer Institute..

[7] Gandhi S, Khubchandani S, Iyer R (2014). Quality of life and hepatocellular carcinoma. Journal of gastrointestinal oncology..

[8] Bonnetain F, Paoletti X, Collette S, Doffoel M, Bouché O, Raoul JL (2008). Quality of life as a prognostic factor of overall survival in patients with advanced hepatocellular carcinoma: Results from two French clinical trials. Quality of life research : An international journal of quality of life aspects of treatment, care and rehabilitation..

[9] Li L, Mo FK, Chan SL, Hui EP, Tang NS, Koh J (2017). Prognostic values of EORTC QLQ-C30 and QLQ-HCC18 index-scores in patients with hepatocellular carcinoma - clinical application of health-related quality-of-life data. BMC Cancer.

[10] Diouf M, Filleron T, Barbare JC, Fin L, Picard C, Bouché O (2013). The added value of quality of life (QoL) for prognosis of overall survival in patients with palliative hepatocellular carcinoma. Journal of hepatology..

[11] Wible BC, Rilling WS, Drescher P, Hieb RA, Saeian K, Frangakis C (2010). Longitudinal quality of life assessment of patients with hepatocellular carcinoma after primary transarterial chemoembolization. Journal of Vascular and Interventional Radiology.

[12] Chie WC, Blazeby JM, Hsiao CF, Chiu HC, Poon RT, Mikoshiba N (2012). International cross-cultural field validation of an european organization for research and treatment of cancer questionnaire module for patients with primary liver cancer, the european organization for research and treatment of cancer quality-of-life questionnaire HCC18. Hepatology.

[13] Yang Z, Wan C, Li W, Cun Y, Meng Q, Ding Y (2015). Development and validation of the simplified chinese version of EORTC QLQ-HCC18 for patients with hepatocellular carcinoma. Cancer investigation..

[14] Piena MA, Schoeman O, Palace J, Duddy M, Harty GT, Wong SL (2020). Modified Delphi study of decision-making around treatment sequencing in relapsing–remitting multiple sclerosis. European Journal of Neurology..

[15] Palace J, Bregenzer T, Tremlett H, Oger J, Zhu F, Boggild M (2014). UK multiple sclerosis risk-sharing scheme: a new natural history dataset and an improved Markov model. British Medical Journal Open.

[16] Topalian SL, Hodi FS, Brahmer JR, Gettinger SN, Smith DC, McDermott DF (2012). Safety, activity, and immune correlates of Anti–PD-1 antibody in cancer. New England Journal of Medicine..

[17] Bersanelli M, Leonetti A, Buti S (2017). The link between calcitriol and anticancer immunotherapy: Vitamin D as the possible balance between inflammation and autoimmunity in the immune-checkpoint blockade. Immunotherapy.

[18] Oken MM, Creech RH, Tormey DC, Horton J, Davis TE, McFadden ET (1982). Toxicity and response criteria of the eastern cooperative oncology group. American Journal of Clinical Oncology..

[19] Aaronson NK, Ahmedzai S, Bergman B, Bullinger M, Cull A, Duez NJ (1993). The European organization for research and treatment of cancer QLQ-C30: A quality-of-life instrument for use in international clinical trials in oncology. Journal of the National Cancer Institute..

[20] Kemmler G, Holzner B, Kopp M, Dünser M, Margreiter R, Greil R (1999). Comparison of two quality-of-life instruments for cancer patients: The functional assessment of cancer therapy-general and the European organization for research and treatment of cancer quality of life questionnaire-C30. Journal of Clinical Oncology : Official Journal of the American Society of Clinical Oncology..

[21] Blazeby JM, Currie E, Zee BC, Chie WC, Poon RT, Garden OJ (2004). Development of a questionnaire module to supplement the EORTC QLQ-C30 to assess quality of life in patients with hepatocellular carcinoma, the EORTC QLQ-HCC18. European Journal of Cancer.

[22] Clinical Trial Endpoints for the Approval of Cancer Drugs and Biologics U.S. Department of Health and Human Services Food and Drug Administration; 2018.

[23] Hettle R, Harty G, Wong SL (2018). Cost-effectiveness of cladribine tablets, alemtuzumab, and natalizumab in the treatment of relapsing-remitting multiple sclerosis with high disease activity in England. Journal of Medical Economics.

[24] Cronbach L (1951). Coefficient alpha and the internal structure of tests. Psychometrika.

[25] Shrout PE, Fleiss JL (1979). Intraclass correlations: Uses in assessing rater reliability. Psychological Bulletin..

[26] Terwee CB, Bot SD, de Boer MR, van der Windt DA, Knol DL, Dekker J (2007). Quality criteria were proposed for measurement properties of health status questionnaires. Journal of Clinical Epidemiology..

[27] National Institute for Health and Care Excellence (2007) Adalimumab, etanercept and infliximab for the treatment of rheumatoid arthritis 40773587

[28] Choo SP, Tan WL, Goh BKP, Tai WM, Zhu AX (2016). Comparison of hepatocellular carcinoma in Eastern versus Western populations. Cancer.

[29] Liang MH (2000). Longitudinal construct validity: establishment of clinical meaning in patient evaluative instruments. Medical Care..

[30] Olejnik S, Algina J (2003). Generalized eta and omega squared statistics: Measures of effect size for some common research designs. Psychological Methods..

[31] Chie WC, Blazeby JM, Hsiao CF, Chiu HC, Poon RT, Mikoshiba N (2017). Differences in health-related quality of life between European and Asian patients with hepatocellular carcinoma. Asia-Pacific Journal of Clinical Oncology..

[32] Osoba D, Rodrigues G, Myles J, Zee B, Pater J (1998). Interpreting the significance of changes in health-related quality-of-life scores. Journal of Clinical Oncology : Official Journal Of The American Society Of Clinical Oncology..

[33] King MT (1996). The interpretation of scores from the EORTC quality of life questionnaire QLQ-C30. Quality of Life Research : An International Journal Of Quality Of Life Aspects Of Treatment, Care And Rehabilitation..

[34] Spearman C (1910). Correlation calculated from faulty data. British Journal of Psychology 1904–1920.

[35] Brown W (1910). Some experimental results in the correlation of mental abilities. British Journal of Psychology 1904–1920.

